# Molybdate in Rhizobial Seed-Coat Formulations Improves the Production and Nodulation of Alfalfa

**DOI:** 10.1371/journal.pone.0170179

**Published:** 2017-01-18

**Authors:** Jiqiong Zhou, Bo Deng, Yingjun Zhang, Adam B. Cobb, Zhao Zhang

**Affiliations:** 1 Department of Grassland Science, College of Animal Science & Technology, China Agricultural University, Beijing, China; 2 Oklahoma State University, Stillwater, Oklahoma, United States of America; 3 Institute of Agricultural Resources and Regional Planning, Chinese Academy of Agricultural Science, Beijing, China; Wuhan Botanical Garden, CHINA

## Abstract

Rhizobia-legume symbiosis is the most well researched biological nitrogen fixation system. Coating legume seeds with rhizobia is now a recognized practical measure for improving the production of legume corp. However, the efficacy of some commercial rhizobia inoculants cannot be guaranteed in China due to the low rate of live rhizobia in these products. A greenhouse experiment was conducted to assess the effects of different rhizobial inoculant formulations on alfalfa productivity and nitrogen fixation. Two rhizobia strains, (ACCC17631 and ACCC17676), that are effective partners with alfalfa variety Zhongmu No. 1 were assessed with different concentrations of ammonium molybdate in seed-coat formulations with two different coating adhesives. Our study showed that the growth, nodulation, and nitrogen fixation ability of the plants inoculated with the ACCC17631 rhizobial strain were greatest when the ammonium molybdate application was0.2% of the formulation. An ammonium molybdate concentration of 0.1% was most beneficial to the growth of the plants inoculated with the ACCC17676 rhizobial strain. The sodium carboxymethyl cellulose and sodium alginate, used as coating adhesives, did not have a significant effect on alfalfa biomass and nitrogen fixation. However, the addition of skimmed milk to the adhesive improved nitrogenase activity. These results demonstrate that a new rhizobial seed-coat formulation benefitted alfalfa nodulation and yield.

## Introduction

Alfalfa (*Medicago sativa* L.) is a leading forage species with wide distribution and the largest cultivated acreage in China [1, 2]. As perennial leguminous forage with access to fixed atmospheric nitrogen (N_2_), alfalfa has a long history in livestock production and grassland restoration due to its high nutritional value. In recent years, the Chinese dairy industry has been shaken by product-quality scandals that are mainly due to the lack of high-quality protein in cattle feed. Therefore, alfalfa production can play a critical role in improving dairy quality as well as the success of China’s dairy industry. However, because of its small-size seed and limited seed nutrients, alfalfa can be negatively impacted by unfavorable germination conditions such as drought and cold. Seed-coat products are increasingly recognized to improve seed germination and increase alfalfa production.

Rhizobia-legume symbiosis is the most well researched biological nitrogen fixation system. It has been proved that rhizobia can increase host-plant nitrogen supply by colonizing its roots and exchanging nitrogen fixed by the bacteria for plant photosynthate within root nodules [3, 4]. Coating alfalfa seeds with rhizobia inoculation products can also increase seed size, making them uniformly shaped and protected from certain pests. This is conducive to mechanized planting, as well as improving nitrogen fixation and alfalfa yield [5].

There is typically limited success from coating seeds with rhizobia because it is difficult to maintain living and active bacterial cells. The efficacy of some commercial rhizobia inoculants cannot be guaranteed in China due to the low rate of live rhizobia in these products [6, 7]. Factors such as temperature, humidity, and toxic substances all affect the survival of rhizobia in the seed-coating agent [8]. As an essential component of nitrate reductase and nitrogenase, molybdenum (Mo) plays a central role in nitrogen metabolism [9]. Studies have found molybdenum fertilizer can significantly increase the activities of nitrogenase, glutamine synthetase and asparagine synthetase, enhancing symbiotic N_2_ fixation capacity of root nodules and the nitrogen metabolism of plants [10]. Thus, adding an appropriate amount of molybdenum to the inoculation formulas could improve seed respiration and increase the survival of the rhizobial cells [11]. However, an overabundance of Mo would change the permeability of bacteroid cell membranes and prevent the normal transportation of ammonia. This could inhibit nitrogenase activity, resulting in reduced nitrogen fixation in root nodules [12]. Moreover, it has been suggested that the responses of different strains of rhizobia to Mo fertilization are dissimilar and depend on various factors, especially Mo concentration in the seed-coating formulation [11, 13]. Utilizing an adhesive agent in seed-coat inoculants enables the rhizobial carrier medium to attach to seeds and prevents or diminishes the direct threat of substances that could potentially contaminate the rhizobia on the seed surface [14]. Researchers report rhizobia activity can be significantly influenced by different adsorbent substances [15, 16]. Due to low cost and environmental safety, both carboxymethyl cellulose (CMC) and alginate (AER) are the most common polymeric material, used for commercial microorganism inoculation. These polymers have been demonstrated to protect the rhizobia against adverse environmental conditions and retain a large number of viable cells even after six months of storage [15, 17, 18]. Therefore, it is critical to assess new seed-coat formulations to determine their potential quality and efficiency for rhizobial inoculation. The objectives of this study were to investigate the effect of different concentrations of ammonium molybdate and different adhesive agents on the activity of rhizobia in seed-coat inoculant formulations as well as potential impacts on alfalfa biomass. These assessments will provide technical support for achieving high yields of high-quality alfalfa in agricultural production.

## Materials and Methods

### Screening for host-rhizobia symbiosis

The study was performed in China Agricultural University, Beijing, China. A hydroponic pre-experiment was conducted to screen for the best host-rhizobia symbiosis using alfalfa variety Zhongmu No. 1. Five fast-growing *Rhizobia meliloti* strains ACCC17537, ACCC17558, ACCC17617, ACCC17631 and ACCC17676 were obtained from the Agricultural Culture Collection of China (ACCC). The bacteria were activated in 250-mL flasks containing 100 mL yeast extract-mannitol (YMA) on a rotary shaker at constant rotation of 150 rpm for 48 h at 28°C.

Alfalfa (Zhongmu No. 1) seeds were soaked in 95% ethanol for 5 min, surface sterilized with bromogeramine for 5 min and then washed with sterile water 10 times. The sterilized seeds were sown in Petri dishes and covered with sterile wet filter paper, then placed in a 25°C incubator to germinate for 48 h. The successfully germinated seeds were soaked in the activated inoculum for 30 min. Alfalfa seedlings were transplanted and fixed on a filter paper bridge in a 1.8 cm×18 cm test tube containing 22ml McKnight's nutrient solution [19, 20]. 2 mL of liquid culture grown to exponential phase or 2 ml of rhizobia-free media control were added to each tube before transplanting. The test tubes were cultured in a 25°C (± 1°C) incubator under a 14 h/10 h light/dark cycle.

Each treatment (5 strains and 1 rhizobia-free control) was replicated six times and the root-position of nodulation, nodulation rate, height and biomass of the plants were observed and measured after 10 weeks of growth.

### The tolerance of rhizobia to molybdenum

Two strains showing the best host-rhizobia symbiosis with Zhongmu No. 1 were selected to assess the tolerance of the rhizobia to molybdenum. Sterilized solid YMA media were prepared with seven ammonium molybdate levels (W/V: 0%, 0.05%, 0.1%, 0.2%, 0.3%, 0.4% and 0.5%). Rhizobia strains were scraped and washed with 9 mL of sterile water then inoculated on the solid YMA media with 0.1 mL of 10^−6^ diluted culture solution. The survival of rhizobia was determined with the viable plate count method [21] at 24 h, 48 h or 72 h after inoculation.

### Assessing new rhizobia seed-coat formulations

A greenhouse experiment was conducted to examine the effect of different rhizobia seed-coat formulations on the growth of alfalfa. This study was a completely randomized 4×3 factorial design with 9 replications. Based on the tolerance of these two rhizobia strains to Mo, there were four levels of ammonium molybdate addition (W/V):0, 0.1%, 0.2% and 0.3% for the strain ACCC17631, and 0, 0.05%, 0.1% and 0.2% for the strain ACCC17676. Adhesive agents were tested at three levels: sodium carboxymethyl cellulose, sodium alginate and sodium alginate + skimmed milk (Devondale, Australian). For simplicity, we abbreviate carboxymethyl cellulose as CMC, sodium alginate as AE and sodium alginate + skimmed milk as AES in this study. Skimmed milk has been shown to act as a protective agents and preserve rhizobia [22]. In this study, skimmed milk was prepared as 10% (W/V) of the bacterial culture prior to seed-coating [23].

Ammonium molybdate was enriched in autoclaved peat adsorbent formulation [24], that was combined with liquid rhizobia at a ratio of 1:2 for a solid bacterial fertilizer (~ 5×10^9^ rhizobia per g to ensure that there were no less than 10^3^ effective rhizobia on the surface of each seed). 40–50 mL 4% adhesive agent was added to 100 g of solid rhizobia inoculant. Detailed formulation is shown in Table 1. Surface-sterilized seeds (1000g) were treated with each factorial combination of rhizobial strain, molybdate fertilizer concentration, and adhesive agent, and then stirred thoroughly to ensure each seed was wrapped with a uniform layer of coated materials. The coated seeds were dried and stored in a ventilated and sterile environment.

**Table 1:** The seed coated formulations.

Treatment	ACCC17631	ACCC17676
No. 1	(A1) Mo 0% + CMC	(B1) Mo 0% + CMC
No. 2	(A2) Mo 0% + AE	(B2) Mo 0% + AE
No. 3	(A3) Mo 0% + AES	(B3) Mo 0% + AES
No. 4	(A4) Mo 0.1% + CMC	(B4) Mo 0.05% + CMC
No. 5	(A5) Mo 0.1% + AE	(B5) Mo 0.05% + AE
No. 6	(A6) Mo 0.1% + AES	(B6) Mo 0.05% + AES
No. 7	(A7) Mo 0.2% + CMC	(B7) Mo 0.1% + CMC
No. 8	(A8)Mo 0.2% + AE	(B8)Mo 0.1% + AE
No. 9	(A9) Mo 0.2% + AES	(B9) Mo 0.1% + AES
No. 10	((A10) Mo 0.3% + CMC	(B10) Mo 0.2% + CMC
No. 11	(A11) Mo 0.3% + AE	(B11) Mo 0.2% + AE
No. 12	(A12) Mo 0.3% + AES	(B12) Mo 0.2% + AES

The factorial combinations of rhizobial strains, molybdate fertilizer concentrations, and adhesive agents. Abbreviations are as follows: Ammonium molybdate concentration (Mo), Carboxymethyl cellulose (CMC), Sodium alginate (AE), Sodium alginate + skimmed milk (AES).

The coated seeds were sown in 1-L nursery pots, filled with clay loam collected from Songzhuang (Tongzhou, Beijing, China), then watered thoroughly. Soil pH was 6.8, and soil contained 11.52 mg kg^−1^ plant-available P, 8.7 mg kg^−1^ Total N, and 10.0 g kg^−1^ soil organic carbon. All soil was steam sterilized at 80°C for 2 h on consecutive days and then dried overnight in a sterile environment to eliminate native microbes. Non-coated seeds (experimental controls) were also prepared. Pots were organized in a completely randomized design and the greenhouse was maintained between 24–27°C. The total number and weight of nodules, the number and weight of effective nodules, nitrogenase activity, and the height and the dry weight of the alfalfa plants were assessed after 45 days of growth. The pink nodules were identified as effective nodules with N-fixing activity and the nodulation rate was calculated as the number of plants with nodulation divided by the total number of plants.

The reduction of acetylene to ethylene was used to detect nitrogenase activity [25–27]. Roots were washed carefully after harvesting; the extra water in the root system was absorbed by filter paper. Nodules on the main root were fully removed, while the nodules on the lateral roots were cut with approximately 0.5 cm of root to retain the activity of the nodules. The collected nodules were placed into a 6-mL reaction vials sealed with rubber stoppers. 0.6 mL of gas was withdrawn from rubber-capped vials, while 0.6 mL acetylene gas was injected. Vials were shaken at 150 rotations per min at 28°C. At the end of the assay, we measured the ethylene present in the gas phase by GC7890F Gas Chromatography (Shanghai Tian Mei, Shanghai, China). The measurement conditions were as follows: column temperature of 180°C, the inlet temperature of 150°C and detector temperature of 170°C. The formula N = hx×C×V/hs×24.9×t [hx, sample peak area; hs, standard C_2_H_4_ peak area; C, standard C_2_H_4_ concentration (μmol/mL); V, the volume of the sample tube; t, C_2_H_2_ reaction time (h); N, C_2_H_4_ concentration (μmol·mL^-1^·h^-1^)] was utilized to calculate the nitrogenase activity of each strain [28].

### Data analysis

One-way analysis of variance (ANOVA) was used to test the best matching symbiosis with Zhongmu No. 1 as well as the tolerance of the two strains, ACCC17631 and ACCC17676 to molybdenum. Two-way ANOVA was employed to test the impacts of Mo, adhesive agents and their interactions on the height, dry weight, number and weight of the nodules and nitrogenase activity of alfalfa. The differences between the adhesive treatments at each level of Mo addition were compared using T-tests (*P* < 0.05). Duncan’s comparison for multiple variables was performed for the different adhesive treatments at various levels of Mo addition (*P* < 0.01). The above statistical analyses were conducted using SAS version 9.1 (SAS Institute, Cary, North Carolina, USA, 2002). All graphs were generated using Sigmaplot version 12.0.

## Results

### Screening for host-rhizobia symbiosis

In the hydroponic experiments, we observed nodules mostly occurred in the lateral roots and there were only a few found in the main root after 10 weeks of growth. Rhizobia inoculation significantly improved nodulation, and there was a significant difference between rhizobial strains (Table 2). The nodulation rates were 100% for ACCC17631 and ACCC17676, while the nodulation rate of plants inoculated with ACCC17558 was 33.33%, which was similar to the non-inoculated control. The total number of nodules and effective nodules also increased significantly for plants inoculated with ACCC17631 or ACCC17676, compared with the non-inoculated control. Inoculation with the other three strains was less effective compared to theACCC17631 and ACCC17676 strains; however, the number of nodules in these treatments was still greater than the non-inoculated control.

**Table 2:** The effect of inoculation with different rhizobia strains on the number of alfalfa nodules and effective nodules.

Strain	Nodulation rate %	Total number of nodules (number/plant)		Number of effective nodules (number/plant)	
		Zhongmu No. 1	Increase compared with the control (%)	Zhongmu No. 1	Increase compared with the control (%)
CK	33.33%	5		1	
17537	66.67%	27	440	22	2,100
17558	33.33%	7	40	7	600
17617	50%	11	120	9	800
17631	100%	36	620	27	2,600
17676	100%	34	580	32	3,100

**Fig 1:**
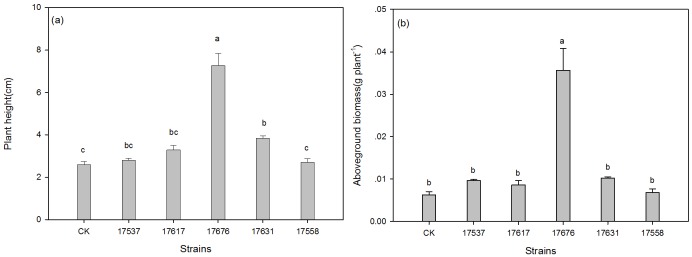
Plant height (a) and aboveground biomass (b) of alfalfa inoculated with different rhizobia.

The height and dry weight of the inoculated alfalfa increased significantly in some cases (Fig 1). The strains ACCC17676 and ACCC17631 significantly increased plant height and biomass. The plants inoculated with ACCC17558 did not result in significant height differences compared to the non-inoculated control. Only plants inoculated with ACCC17676 had significantly increased biomass relative to the control. According to the analysis of growth indicators in the alfalfa plants after inoculation with the five strains, ACCC17676 and ACCC17631 rhizobia showed optimal symbiotic matching with Zhongmu No. 1 alfalfa (S1 Table). Therefore, the rhizobia strains ACCC17676 and ACCC17631 were selected for further use in seed coat experiments.

### The tolerance of rhizobia to molybdenum

The strains ACCC17676 and ACCC17631 displayed different tolerances to Mo addition and ACCC17631 performed better than ACCC17676 at greater Mo concentrations. ACCC17631 was strongly inhibited when Mo concentration reached or surpassed 0.3%, while the growth and proliferation of ACCC17676 cells were suppressed at or above 0.2% Mo application (Table 3, S2 and S3 Tables). Thus, for the seed coating formulation experiment, carriers containing Mo at concentrations of 0.1%, 0.2%, and 0.3% were applied for ACCC17631, and Mo concentrations of 0.05%, 0.1% and 0.2% were added to rhizobia strain ACCC17676.

**Table 3:** Impact of different concentrations of ammonium molybdate on the growth of rhizobia strains.

Strain	Time (h)	Concentrations of ammonium molybdate (%)						
		0	0.05	0.1	0.2	0.3	0.4	0.5
ACCC17631	24	+++++	+++++	+++++	+++++	++++	++	+
	48	+++++	+++++	+++++	+++++	++++	++	++
	72	+++++	+++++	+++++	+++++	++++	++	++
ACCC17676	24	+++++	+++++	+++++	+++	++	+	-
	48	+++++	+++++	+++++	+++	++	+	-
	72	+++++	+++++	+++++	+++	+++	+	-

Plus sign denote a significant preference (*p < 0*.*05*): +++++ Excellent; ++++ Good; +++ Normal; ++ Below normal; + Poor;—No growth.

### Assessing new rhizobia seed-coat formulations

Our results showed the different seed coated formulations (including different concentrations of Mo and adhesive agents) significantly affected the height, aboveground biomass, number of root nodules, weight of nodules and nitrogenase activity of the alfalfa plants (as shown in Table 4).

**Table 4:** Analysis of variance for the effects of different Mo concentrations, adhesive agents and their interactions on plant height, aboveground biomass, the number of nodules, nodule weight and nitrogenase activity in alfalfa.

Treatments	Plant height	Aboveground biomass	Number of nodules	Nodule weight	Nitrogenase activity
Strain ACCC17631					
Mo concentration	**< .0001**	**< .0001**	**< .0001**	**< .0001**	**< .0001**
Adhesive agent	0.2151	0.2491	0.5347	0.8587	**0.0093**
Mo × Adhesive	0.1323	0.6728	0.8701	0.8898	0.7836
Strain ACCC17676					
Mo concentration	**< .0001**	**< .0001**	**< .0001**	**< .0001**	**< .0001**
Adhesive agent	0.6280	0.8923	0.9581	0.2831	**0.0003**
Mo × Adhesive	0.8450	0.9497	0.9384	0.9310	0.1173

**Fig 2:**
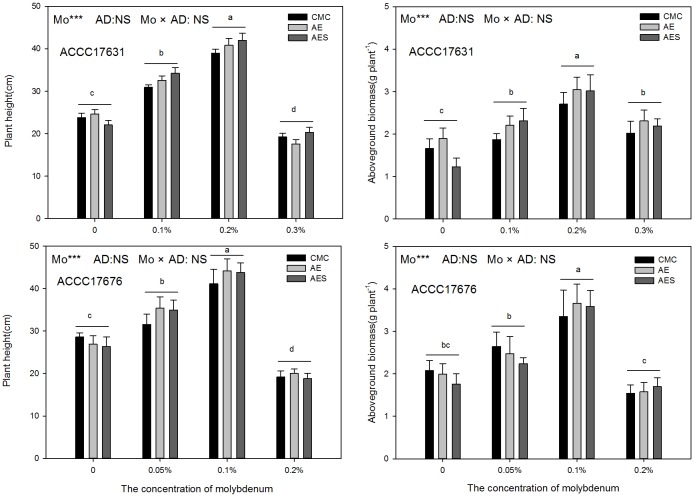
Plant height and aboveground biomass of alfalfa inoculated with two rhizobia seed coat formulations. Between the four Mo concentrations under the control treatment, bars that do not share capital letters are significantly different (*P < 0*.*01*). The data are presented as means ± SE. *** *P < 0*.*001; NS P > 0*.*05*. Abbreviations are as follows: Adhesive agent (AD), Ammonium molybdate concentration (Mo), Carboxymethyl cellulose (CMC), Sodium alginate (AE), Sodium alginate + skimmed milk (AES).

Molybdenum enriched inoculation formulations significantly promoted the growth of Zhongmu No.1 (Fig 2). Plants inoculated with ACCC17631 showed greater height and aboveground biomass with 0.2% Mo addition in comparison to other treatments. When the A9 seed-coat formulation (0.2% Mo and AES) was applied, the height of the alfalfa plants reached a peak of 42.01 cm and the biomass production reached a peak of 3.04 g per plant. Comparatively, plant height and biomass decreased significantly when the concentration of Mo exceeded 0.2% (S4 Table). A similar trend was found for alfalfa inoculated with ACCC17676 rhizobia. Both height and aboveground biomass increased significantly with Mo addition and peaked when Mo comprised 0.1% of the seed coat (S5 Table). Moreover, there were no significant differences in alfalfa height or biomass between different adhesive agents used in seed coat treatments regardless of rhizobia strain. The effect of the interaction between Mo addition and adhesive type was also not significant.

**Fig 3:**
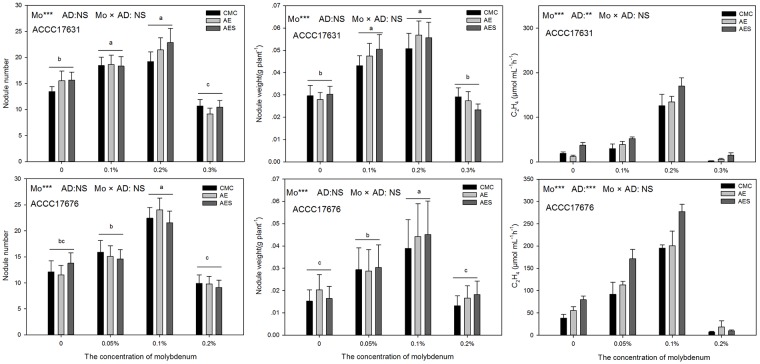
The number, weight and nitrogenase activity of root nodules in alfalfa inoculated with different rhizobia seed coat formulations. Note: Between the four Mo concentrations under the control treatment, bars that do not share a capital letters are significantly different (*P < 0*.*01*). The data are presented as means ± SE. *** *P < 0*.*001*;** *P < 0*.*01; NS P > 0*.*05*. Abbreviations are as follows: Adhesive agent (AD), Ammonium molybdate concentration (Mo), Carboxymethyl cellulose (CMC), Sodium alginate (AE), Sodium alginate + skimmed milk (AES).

Mo additions significantly increased the number and weight of nodules on the roots of alfalfa regardless of rhizobia strain, whereas different adhesives did not affect the number and the weight of the nodules (Fig 3). For the plants inoculated with ACCC17631 rhizobia, the greatest number of nodules at 22.89/plant (A9: 0.2% Mo + AES) and greatest weight of nodules at 0.057 g/plant (A8: 0.2% Mo + AE) were recorded in treatments receiving the 0.2% Mo concentration (S6 Table). Mo additions significantly increased nitrogenase activity in root nodules, compared to control. The activity of nitrogenase was greatest in the nodules of plants coated with formulation A9 (Mo 0.2% + AES), resulting in a C_2_H_4_ concentration of 169.95 μmol·mL^-1^·h^-1^. This C_2_H_4_ concentration was 8.9 times greater than the 19.01 μmol·mL^-1^·h^-1^ recorded for formulation A1 (Mo 0% + CMC). Neither adhesive agent showed a significant impact on nitrogenase activity, but the addition of skimmed milk significantly improved nitrogenase activity in alfalfa roots with formulation A9 (0.2% Mo + AES) increasing activity by 35.24% and 26.21% compared with A7 (0.2% Mo + CMC) and A8 (0.2% Mo + AE) formulations that did not include skimmed milk (S7 Table).

Similarly, the number, weight, and nitrogenase activity in the plants inoculated with ACCC17676 rhizobia were also significantly improved by Mo addition (S8 and S9 Tables). 0.1% Mo was the most beneficial to nodulation as well as nitrogenase activity. For this rhizobia strain, B8 (0.1% Mo + AE) seed coating resulted in the greatest number of root nodules (24 per plant); however, the weight of the root nodules and the nitrogenase activity were greatest with the B9 (0.1% Mo + AES) formulation.

## Discussion

When considering the inoculation of legume crops with rhizobia, the first step is finding a highly effective strain. *R*. *meliloti* ACCC17631 and ACCC17676, isolated from Xinjiang and Shandong province in China and cultured with alfalfa, were effective partners for Zhongmu No.1 alfalfa. Our results indicate ACCC17676 was more beneficial to aboveground plant production and root nodulation, as well as nitrogenase activity than inoculation with ACCC17631, however, ACCC17631 has been shown to have more environmental adaptability and higher tolerance for adverse conditions [29]. We also tested the stress resistance of these two strains. ACCC17676 is more sensitive to salt and high pH, whereas the growth rates of ACCC17631 relatively unaffected by salt or alkali with NaCl concentration of culture media up to 7.0% (W/V) or culture pH up to 9.5. Therefore, both of these strains would be the suitable candidates for commercial rhizobial inoculation production and should be chosen for different specific soil conditions and properties.

Typically, legumes have small Mo requirements but low Mo availability can inhibit nitrogenase activity in root nodules [30]. The benefits of sowing seeds enriched with Mo include increasing plant yields, as shown for common beans [31–33] and soybeans [11]. Our greenhouse study demonstrated that 0.1%-0.2% molybdenum supplied in the rhizobia seed-coat formulations significantly increased the plant heights and aboveground biomasses for both rhizobia strains. This agrees with Rahman et al. (2008) who reported that mungbeans exhibit superior yield when they combined rhizobia inoculant along with 1.0 kg Mo/ha in silty clay loam soils [34] and Campo et al. (2009) who demonstrated inoculation of Mo-rich seeds significantly increased soybean yield and total N compared to the non-inoculated seeds and control [11]. The majority of existing research focuses on the single effect of Mo seed enrichment of leguminous crops [35], or the root nodulation response to supplemental Mo fertilizer with peat-based powders or liquid inoculants [36]. The development and impact of applying Mo directly into seed-coat inoculations have received much less attention, particularly for alfalfa. The reason may be that plant seeds and rhizobia are susceptible to Mo overdosing, which could reduce the survival of rhizobia, damage seed respiration, and decrease plant nodulation and N_2_ fixation [37, 38]. These toxic effects of Mo depend on the plant species and strains of microbes, soil pH, and other environmental factors [39–42]. Therefore, the Mo tolerance threshold of different rhizobia strains and crops should be assessed carefully. Our research showed rhizobia ACCC17631 grew well with Mo content below 0.4% but ACCC17676 cells were suppressed with Mo content at or above 0.2%. Further testing revealed that the growth and nodulation of alfalfa were strongly inhibited when Mo concentration exceeded the rhizobia tolerance threshold content. Similar results have been reported when high concentrations of Mo had negative osmotic effects on Bradyrhizobium [42, 43] and an inhibitory effect on the activity of phosphatase in pea plumules [44, 45]. However, legume crop yield and nodulation did not always respond to the addition of Mo to seed inoculant formulas. No significant effects were observed due to the Mo content of black beans, common beans, and soybeans [13, 35, 46] These different observations most likely relate to differences in plant genetics with regard to Mo accumulation in seeds [47].

During the last two decades, polymeric materials were evaluated as potential bacterial carriers or seed inoculant adhesive agents due to their low cost, strong compatibility with bacteria, and ease of field application [48–50]. CMC and AE are the most common high molecular synthetic compounds for entrapped microbes [51, 52], can stick closely to coated materials, sustain live rhizobial cells, are harmless to cotyledons, and are not toxic in the environment [53, 54]. While many reports have focused on the ability of polymers to sustain rhizobia on legume seeds, especially on cowpea and soybean seeds [49, 55–58], their effects on root nodulation and N-fixation have not been well quantified. CMC is a cellulose-derived ester and pre-studies indicated that CMC has proper chemistry characteristics for stable microbe storage [48, 59, 60]. Alginate is a naturally formed polymer and wildly used for entrapping microorganisms in two main forms, encapsulated formulations and alginate beads [61]. Alginate encapsulation and alginate beads have been verified to improve microorganism survival time, but alginate products are difficult to blend with seeds for commercial application. The bacteria released from the beads must migrate through the soil, compete with native microflora, and strive to attach to seeds [62]. Our study explored a new way to compare the effects of different polymers as the adhesive agent for seed-coat inoculations. Although no significant differences were observed in plant height, biomass production, nodule number, or nodule weight between the adhesive agents, the activity of nitrogenase improved significantly when skimmed milk was added to the AE-adhesive. Nitrogenase activity within root nodules is typically the limiting factor for N-fixation in legume crops [63]. Alginate formulations containing skimmed milk have effectively conserved the viability of many Gram-negative bacteria [64, 65]. The highest experimental survival of Azosprillum was achieved by the addition of skim milk to Alginate beads, with greater than 88% survival even after 150 days of storage [66]. Nitrogenase activity would be linked with rhizobium effectiveness, active nodule number, and nodule weight. Vieira et al. (1998) reported that more effective rhizobium had a positive influence on nitrogenase activity [67]. In our study, nitrogenase activity improved in the alginate + skimmed milk treatment, which may be as a result of increased adhesive agent protection of live rhizobial cells.

It is a slow process to develop new and effective rhizobial seed-coat inoculant formulations. Rhizobium inoculation may function ideally under precise greenhouse conditions, with scientific equipment and the management of technical personnel, but it is difficult to achieve similar results for an effective product used in field conditions by microbiologically untrained farmers [68]. The introduced bacteria need to find an empty niche in the soil and compete with the well-adapted native microflora. Generally, the industry procedures for developing microbial inoculants will include the following steps: isolation and screening of the best rhizobial strain, identification of the strains, formulation, growth chamber and greenhouse testing, field microplot testing, release to industry, mass production, testing in farmer’s fields, registration and commercialization, and finally wide field use [61]. We recognize our work is the beginning of a lengthy process and many issues will need to be addressed; such as, how we can improve the survival of rhizobia in the formulation, how we can ensure good field performance of seed-coat inoculants, how we can develop low-cost technologies for extending the shelf life of inoculation products. Formulation and field function of inoculants are a matter of sustainable agriculture and environmental development rather than merely a technical challenge. Further studies will be conducted to assess the benefits of the seed coat formulations in different soil conditions and explain the potential microbiological and material science mechanisms.

## Conclusion

Mo-enriched rhizobial seed-coat inoculants significantly improved alfalfa plant heights, dry weight, root nodule number, nodule weight, and nitrogenase activity. The best Mo concentration for rhizobia strain ACCC17631 is 0.2% (W/V) and rhizobia strain ACCC17676 was most effective at 0.1% (W/V) Mo addition. CMC and AE were applied as adhesive agents to combine solid inoculate and seeds and had no significant effect on plant growth and nodulation. Surprisingly, nitrogenase activity was enhanced significantly by adding skimmed milk into AE adhesive. Overall, the rhizobia seed-coat formulations, which included 0.1% (0.2%) Mo addition and used alginate + skimmed milk as adhesive, were beneficial to alfalfa production and N-fixation in this greenhouse study.

## Supporting Information

S1 Table Plant heights and aboveground biomass of alfalfa inoculated with different rhizobia. (PDF)

S2 Table Impact of different concentrations of ammonium molybdate on the growth of rhizobia strains ACCC17631 (×10^6^ rhizobia). (PDF)

S3 Table Impact of different concentrations of ammonium molybdate on the growth of rhizobia strains ACCC17676 (×10^6^ rhizobia). (PDF)

S4 Table Plant height and aboveground biomass of alfalfa inoculated with ACCC17631 rhizobia seed-coat formulation. (PDF)

S5 Table Plant height and aboveground biomass of alfalfa inoculated with ACCC17676 rhizobia seed-coat formulation. (PDF)

S6 Table The number and weight of root nodules in alfalfa inoculated with ACCC17631 rhizobia seed-coat formulation. (PDF)

S7 Table The nitrogenase activity of root nodules in alfalfa inoculated with ACCC17631 rhizobia seed-coat formulation. (PDF)

S8 Table The number and weight of root nodules in alfalfa inoculated with ACCC17676 rhizobia seed-coat formulation. (PDF)

S9 Table The nitrogenase activity of root nodules in alfalfa inoculated with ACCC17676 rhizobia seed-coat formulation. (PDF)
