# Supplementary material for: Molybdate in Rhizobial Seed-Coat Formulations Improves the Production and Nodulation of Alfalfa
Source: PLoS One. 2017 Jan 18;12(1):e0170179. doi: 10.1371/journal.pone.0170179 (PMC5242510; doi:10.1371/journal.pone.0170179)
Supplement: S1 Table — (PDF) [file pone.0170179.s001.pdf]

S1 Table. Plant heights and aboveground biomass of alfalfa inoculated with different rhizobia.

Plant height(cm)	Rpt.1	Rpt.2	Rpt.3	Rpt.4	Rpt.5	Rpt.6
CK	2.1	2.9	2.9	2.9	2.1	2.6
ACCC17537	2.9	2.8	2.9	3.1	2.6	2.5
ACCC17617	4.1	2.7	3.7	3.2	2.9	3.1
ACCC17676	9.7	7.1	6.2	8.1	6.6	5.9
ACCC17631	3.9	3.7	4.3	3.7	4	3.4
ACCC17558	2.9	2.9	2.3	2.8	2.1	3.2
Biomass(g/plant)	Rpt.1	Rpt.2	Rpt.3	Rpt.4	Rpt.5	Rpt.6
CK	0.0055	0.0085	0.0082	0.0049	0.0041	0.0066
ACCC17537	0.0094	0.0095	0.0095	0.0088	0.0109	0.0099
ACCC17617	0.0132	0.0088	0.0076	0.0089	0.0059	0.0072
ACCC17676	0.0532	0.0497	0.0312	0.0291	0.0224	0.0282
ACCC17631	0.0099	0.0113	0.0105	0.0091	0.0095	0.0109
ACCC17558	0.0083	0.0051	0.0063	0.0068	0.0044	0.0101
