# Supplementary material for: Molybdate in Rhizobial Seed-Coat Formulations Improves the Production and Nodulation of Alfalfa
Source: PLoS One. 2017 Jan 18;12(1):e0170179. doi: 10.1371/journal.pone.0170179 (PMC5242510; doi:10.1371/journal.pone.0170179)
Supplement: S2 Table — (PDF) [file pone.0170179.s002.pdf]

S2 Table. Impact of different concentrations of ammonium molybdate on the growth of rhizobia strains ACCC17631 (×10^6 rhizobia).

24h	Mo concentration (%)	Rpt.1	Rpt.2	Rpt.3	Rpt.4	Rpt.5
	0	52	42	41	76	34
	0.05	42	57	65	73	62
	0.1	69	72	47	51	54
	0.2	55	59	64	50	52
	0.3	20	27	38	45	19
	0.4	9	4	8	11	4
	0.5	0	0	1	0	0
48h	Mo concentration (%)	Rpt.1	Rpt.2	Rpt.3	Rpt.4	Rpt.5
	0	198	183	193	171	204
	0.05	202	205	197	172	166
	0.1	208	179	155	199	184
	0.2	181	184	209	202	172
	0.3	54	85	93	101	84
	0.4	17	15	15	20	8
	0.5	5	0	2	2	0
72h	Mo concentration (%)	Rpt.1	Rpt.2	Rpt.3	Rpt.4	Rpt.5
	0	211	191	219	182	214
	0.05	218	224	229	189	175
	0.1	224	187	173	206	209
	0.2	183	208	223	203	194
	0.3	74	92	99	114	103
	0.4	19	15	18	21	12
	0.5	6	0	4	2	0
