# Supplementary material for: Molybdate in Rhizobial Seed-Coat Formulations Improves the Production and Nodulation of Alfalfa
Source: PLoS One. 2017 Jan 18;12(1):e0170179. doi: 10.1371/journal.pone.0170179 (PMC5242510; doi:10.1371/journal.pone.0170179)
Supplement: S3 Table — (PDF) [file pone.0170179.s003.pdf]

S3 Table. Impact of different concentrations of ammonium molybdate on the growth of rhizobia strains ACCC17676 (×10^6 rhizobia).

24h	Mo concentration (%)	Rpt.1	Rpt.2	Rpt.3	Rpt.4	Rpt.5
	0	28	32	31	50	66
	0.05	31	47	57	30	24
	0.1	44	34	31	32	50
	0.2	29	13	28	10	15
	0.3	6	11	17	8	16
	0.4	3	4	0	8	1
	0.5	0	0	0	0	0
48h	Mo concentration (%)	Rpt.1	Rpt.2	Rpt.3	Rpt.4	Rpt.5
	0	148	138	103	110	145
	0.05	145	127	133	123	127
	0.1	147	129	133	97	109
	0.2	51	45	40	34	55
	0.3	22	15	20	22	20
	0.4	5	7	2	10	4
	0.5	1	0	1	0	0
72h	Mo concentration (%)	Rpt.1	Rpt.2	Rpt.3	Rpt.4	Rpt.5
	0	151	159	127	136	147
	0.05	166	144	152	139	131
	0.1	156	162	137	126	135
	0.2	71	58	56	62	49
	0.3	49	48	52	41	31
	0.4	17	11	6	15	12
	0.5	1	0	3	0	0
