# Supplementary material for: Molybdate in Rhizobial Seed-Coat Formulations Improves the Production and Nodulation of Alfalfa
Source: PLoS One. 2017 Jan 18;12(1):e0170179. doi: 10.1371/journal.pone.0170179 (PMC5242510; doi:10.1371/journal.pone.0170179)
Supplement: S4 Table — (PDF) [file pone.0170179.s004.pdf]

S4 Table. Plant height and aboveground biomass of alfalfa inoculated with ACCC17631 rhizobia seed-coat formulation.

Plant height (cm)	Rpt.1	Rpt.2	Rpt.3	Rpt.4	Rpt.5	Rpt.6	Rpt.7	Rpt.8	Rpt.9
(A1) Mo 0% + CMC	22.1	24.9	27.6	20.1	20.2	22	28.5	26.1	22.8
(A2) Mo 0% + AE	28.2	26.1	29.4	23.2	21.6	21.7	21.9	27.5	22.1
(A3) Mo 0% + AES	27.1	22.6	20.4	23.5	25.9	19.5	20.8	22	16.9
(A4) Mo 0.1% + CMC	29.2	27.9	33.5	31.2	29.4	32	32.7	31.5	30.9
(A5) Mo 0.1% + AE	35.6	31	37.4	27.1	29.3	31.9	31.5	34.6	34.4
(A6) Mo 0.1% + AES	39	33.5	40	31.1	37	35.3	30.4	28.5	33.2
(A7) Mo 0.2% + CMC	42	35.5	40.7	36.3	42.3	39	36.7	35.8	42.4
(A8) Mo 0.2% + AE	40.4	33.2	39	42.4	50	41.7	38.5	44	38.7
(A9) Mo 0.2% + AES	39.4	41	37.2	43	49.2	36.8	50.1	38.1	43.3
(A10) Mo 0.3% + CMC	21.9	18.7	22.9	16.9	15.4	20.1	16.6	19.6	20.9
(A11) Mo 0.3% + AE	15	20.6	20.1	13.8	13.6	19.4	15.4	19.7	20.9
(A12) Mo 0.3% + AES	16.2	14.6	20	20.2	24.3	20.8	19.3	25.8	21.7
Aboveground biomass(g/plant)	Rpt.1	Rpt.2	Rpt.3	Rpt.4	Rpt.5	Rpt.6	Rpt.7	Rpt.8	Rpt.9
(A1) Mo 0% + CMC	1.87	1.85	2.12	1.26	3.01	1.94	0.91	0.92	1.04
(A2) Mo 0% + AE	1.99	1.31	1.5	3.3	1.47	2.04	1.13	1.53	2.81
(A3) Mo 0% + AES	0.92	1.09	0.22	1.03	1.11	1.01	1.39	2.12	2.2
(A4) Mo 0.1% + CMC	2.66	1.72	1.58	1.26	2.18	1.68	1.58	2.08	2.11
(A5) Mo 0.1% + AE	2.62	2.14	2.07	2.17	2.32	3.54	2.32	1.34	1.32
(A6) Mo 0.1% + AES	2.04	2.62	2.1	1.45	1.67	2.48	1.62	2.48	4.34
(A7) Mo 0.2% + CMC	2.45	4.18	1.73	3.47	2.74	2.73	3.31	1.98	1.75
(A8) Mo 0.2% + AE	3.23	4.41	2.82	1.94	4.4	2.92	2.12	2.62	2.94
(A9) Mo 0.2% + AES	2.83	4.68	5.01	2.09	2.3	2.31	1.85	3.4	2.69
(A10) Mo 0.3% + CMC	1.58	1.13	1.86	2.52	3.98	2.09	1.38	1.43	2.21
(A11) Mo 0.3% + AE	4.01	2.18	3.05	1.94	2.03	2.17	2.03	1.65	1.78
(A12) Mo 0.3% + AES	2.26	2.08	1.49	2.98	2.92	2.14	1.61	1.92	2.31
