# Supplementary material for: Molybdate in Rhizobial Seed-Coat Formulations Improves the Production and Nodulation of Alfalfa
Source: PLoS One. 2017 Jan 18;12(1):e0170179. doi: 10.1371/journal.pone.0170179 (PMC5242510; doi:10.1371/journal.pone.0170179)
Supplement: S5 Table — (PDF) [file pone.0170179.s005.pdf]

S5 Table. Plant height and aboveground biomass of alfalfa inoculated with ACCC17676 rhizobia seed-coat formulation.

Plant height (cm)	Rpt.1	Rpt.2	Rpt.3	Rpt.4	Rpt.5	Rpt.6	Rpt.7	Rpt.8	Rpt.9
(B1) Mo 0% + CMC	32.5	27.3	29.2	26.4	30.6	31.2	28.4	29.5	22.1
(B2) Mo 0% + AE	30.7	34.2	28.9	34.3	28.2	26.9	18.8	20.2	19.7
(B3) Mo 0% + AES	23	27.6	20.6	23.1	20.6	21.7	40.6	33.1	27.3
(B4) Mo 0.05% + CMC	40.2	36.8	32	18.6	40.4	28	28.5	35.1	24.4
(B5) Mo 0.05% + AE	27.6	43	29.5	32.2	30.9	47	26.6	46.4	35.2
(B6) Mo 0.05% + AES	33	41.5	35.9	38.8	31.6	24.3	28.6	33.4	47.7
(B7) Mo 0.1% + CMC	41.3	40.2	40.5	37	35.8	35.2	68.1	34.7	37.2
(B8) Mo 0.1% + AE	30.4	36.2	48.1	51.2	52.6	38.8	37.1	50.4	52.6
(B9) Mo 0.1% + AES	35	48.7	47.3	48.6	38.2	37.2	45.8	38.2	55
(B10) Mo 0.2% + CMC	19.7	16.8	17.1	15.1	20.4	23.2	14.1	28.5	17.5
(B11) Mo 0.2% + AE	19.4	22.1	14.3	21.6	18.4	25.5	19.1	18.9	20.8
(B12) Mo 0.2% + AES	17.9	25.2	16.7	18.3	23.6	13.4	15.9	20.4	17.7
Aboveground biomass(g/plant)	Rpt.1	Rpt.2	Rpt.3	Rpt.4	Rpt.5	Rpt.6	Rpt.7	Rpt.8	Rpt.9
(B1) Mo 0% + CMC	2.32	1.48	1.62	1.65	2.86	1.48	3.57	1.68	2.02
(B2) Mo 0% + AE	3.87	1.58	1.56	2.17	1.75	2.23	1.68	1.64	1.42
(B3) Mo 0% + AES	1.59	1.21	1.89	1.48	1.43	1.26	3.29	1.02	2.64
(B4) Mo 0.05% + CMC	2.06	4.01	3.96	3.86	1.57	1.62	2.32	1.91	2.51
(B5) Mo 0.05% + AE	4.45	3.85	3.34	1.31	1.46	2.24	1.4	1.45	2.8
(B6) Mo 0.05% + AES	1.73	1.95	2.3	2.56	2.12	2.05	2.96	2.66	1.83
(B7) Mo 0.1% + CMC	4.43	6.4	5.53	2.01	1.54	1.63	4.421	2.41	1.763
(B8) Mo 0.1% + AE	2.67	2.98	2.87	5.35	6.1	1.92	3.21	3.92	3.95
(B9) Mo 0.1% + AES	2.69	3.32	4.29	1.87	4.02	2.13	4.59	4.91	4.47
(B10) Mo 0.2% + CMC	1.44	0.95	1.46	1.08	1.01	1.15	2.05	2.12	2.64
(B11) Mo 0.2% + AE	1.39	1.63	1.41	2.67	0.94	2.69	1.2	1.06	1.22
(B12) Mo 0.2% + AES	2.84	1.27	1.22	1.48	1.75	2.65	1.59	1.2	1.27
