# Supplementary material for: Molybdate in Rhizobial Seed-Coat Formulations Improves the Production and Nodulation of Alfalfa
Source: PLoS One. 2017 Jan 18;12(1):e0170179. doi: 10.1371/journal.pone.0170179 (PMC5242510; doi:10.1371/journal.pone.0170179)
Supplement: S6 Table — (PDF) [file pone.0170179.s006.pdf]

S6 Table. The number and weight of root nodules in alfalfa inoculated with ACCC17631 rhizobia seed-coat formulation.

Nodules number (number/plant)	Rpt.1	Rpt.2	Rpt.3	Rpt.4	Rpt.5	Rpt.6	Rpt.7	Rpt.8	Rpt.9
(A1) Mo 0% + CMC	13	8	18	14	11	15	13	14	15
(A2) Mo 0% + AE	18	5	20	14	16	19	9	17	22
(A3) Mo 0% + AES	14	11	18	17	8	19	23	13	18
(A4) Mo 0.1% + CMC	18	21	9	21	14	16	19	25	23
(A5) Mo 0.1% + AE	24	15	18	23	10	26	13	21	18
(A6) Mo 0.1% + AES	21	14	15	17	8	20	24	26	20
(A7) Mo 0.2% + CMC	17	28	14	19	26	20	12	14	23
(A8) Mo 0.2% + AE	26	24	10	17	15	25	23	34	19
(A9) Mo 0.2% + AES	21	26	28	14	33	31	14	11	28
(A10) Mo 0.3% + CMC	13	9	8	15	4	11	16	8	12
(A11) Mo 0.3% + AE	7	6	14	11	15	8	9	5	7
(A12) Mo 0.3% + AES	8	12	6	14	18	9	6	12	9
Nodule weight(g/plant)	Rpt.1	Rpt.2	Rpt.3	Rpt.4	Rpt.5	Rpt.6	Rpt.7	Rpt.8	Rpt.9
(A1) Mo 0% + CMC	0.024	0.035	0.017	0.024	0.011	0.048	0.034	0.052	0.022
(A2) Mo 0% + AE	0.033	0.029	0.021	0.043	0.031	0.014	0.016	0.038	0.026
(A3) Mo 0% + AES	0.017	0.048	0.015	0.029	0.034	0.038	0.036	0.025	0.031
(A4) Mo 0.1% + CMC	0.043	0.036	0.03	0.055	0.068	0.025	0.051	0.044	0.036
(A5) Mo 0.1% + AE	0.039	0.044	0.048	0.072	0.027	0.053	0.021	0.068	0.055
(A6) Mo 0.1% + AES	0.066	0.068	0.053	0.022	0.028	0.036	0.08	0.062	0.039
(A7) Mo 0.2% + CMC	0.051	0.068	0.018	0.026	0.049	0.038	0.074	0.058	0.075
(A8) Mo 0.2% + AE	0.044	0.061	0.055	0.077	0.081	0.066	0.024	0.035	0.068
(A9) Mo 0.2% + AES	0.034	0.061	0.069	0.088	0.074	0.026	0.047	0.065	0.037
(A10) Mo 0.3% + CMC	0.037	0.033	0.028	0.015	0.01	0.021	0.042	0.028	0.048
(A11) Mo 0.3% + AE	0.029	0.044	0.008	0.017	0.023	0.028	0.019	0.047	0.031
(A12) Mo 0.3% + AES	0.031	0.009	0.027	0.025	0.034	0.015	0.018	0.027	0.024
