# Supplementary material for: Molybdate in Rhizobial Seed-Coat Formulations Improves the Production and Nodulation of Alfalfa
Source: PLoS One. 2017 Jan 18;12(1):e0170179. doi: 10.1371/journal.pone.0170179 (PMC5242510; doi:10.1371/journal.pone.0170179)
Supplement: S7 Table — (PDF) [file pone.0170179.s007.pdf]

S7 Table. The nitrogenase activity of root nodules in alfalfa inoculated with ACCC17631 rhizobia seed-coat formulation.

Nitrogenase activity(μmol ml^{-1} h^{-1})	Rpt.1	Rpt.2	Rpt.3
(A1) Mo 0% + CMC	25.18751	18.75702	13.09512
(A2) Mo 0% + AE	6.532397	17.29682	12.28373
(A3) Mo 0% + AES	27.42983	37.78986	47.70957
(A4) Mo 0.1% + CMC	16.08622	20.91178	50.96859
(A5) Mo 0.1% + AE	34.11847	53.49004	29.476
(A6) Mo 0.1% + AES	44.64423	56.19938	55.96834
(A7) Mo 0.2% + CMC	73.71265	146.3895	156.882
(A8) Mo 0.2% + AE	128.0482	116.9236	159.0181
(A9) Mo 0.2% + AES	187.9591	189.776	132.1144
(A10) Mo 0.3% + CMC	1.947506	3.483609	0.921512
(A11) Mo 0.3% + AE	2.299614	6.075431	8.831103
(A12) Mo 0.3% + AES	9.973074	26.27035	8.632359
