# Supplementary material for: Molybdate in Rhizobial Seed-Coat Formulations Improves the Production and Nodulation of Alfalfa
Source: PLoS One. 2017 Jan 18;12(1):e0170179. doi: 10.1371/journal.pone.0170179 (PMC5242510; doi:10.1371/journal.pone.0170179)
Supplement: S8 Table — (PDF) [file pone.0170179.s008.pdf]

S8 Table. The number and weight of root nodules in alfalfa inoculated with ACCC17676 rhizobia seed-coat formulation.

Nodules number (number/plant)	Rpt.1	Rpt.2	Rpt.3	Rpt.4	Rpt.5	Rpt.6	Rpt.7	Rpt.8	Rpt.9
(B1) Mo 0% + CMC	6	10	8	13	27	12	16	10	7
(B2) Mo 0% + AE	11	13	8	5	23	13	7	9	15
(B3) Mo 0% + AES	16	14	16	9	5	18	25	8	13
(B4) Mo 0.05% + CMC	17	11	15	9	14	20	28	7	22
(B5) Mo 0.05% + AE	24	15	17	9	14	13	7	25	12
(B6) Mo 0.05% + AES	11	15	6	9	17	23	18	12	20
(B7) Mo 0.1% + CMC	27	20	19	22	13	27	24	17	33
(B8) Mo 0.1% + AE	19	26	32	12	28	16	24	31	28
(B9) Mo 0.1% + AES	15	17	21	35	20	19	14	26	27
(B10) Mo 0.2% + CMC	6	15	20	4	8	10	7	8	11
(B11) Mo 0.2% + AE	5	12	8	6	10	19	13	8	7
(B12) Mo 0.2% + AES	13	9	4	8	12	17	5	7	7
Nodule weight(g/plant)	Rpt.1	Rpt.2	Rpt.3	Rpt.4	Rpt.5	Rpt.6	Rpt.7	Rpt.8	Rpt.9
(B1) Mo 0% + CMC	0.009	0.013	0.017	0.006	0.022	0.028	0.017	0.015	0.01
(B2) Mo 0% + AE	0.023	0.015	0.028	0.007	0.016	0.034	0.028	0.014	0.018
(B3) Mo 0% + AES	0.015	0.004	0.009	0.025	0.022	0.016	0.013	0.032	0.012
(B4) Mo 0.05% + CMC	0.017	0.032	0.03	0.019	0.025	0.024	0.046	0.038	0.033
(B5) Mo 0.05% + AE	0.017	0.028	0.034	0.041	0.019	0.025	0.017	0.036	0.042
(B6) Mo 0.05% + AES	0.026	0.018	0.035	0.015	0.029	0.027	0.041	0.052	0.03
(B7) Mo 0.1% + CMC	0.031	0.042	0.028	0.061	0.037	0.045	0.017	0.053	0.036
(B8) Mo 0.1% + AE	0.052	0.058	0.034	0.042	0.061	0.055	0.028	0.023	0.045
(B9) Mo 0.1% + AES	0.027	0.032	0.049	0.058	0.068	0.051	0.044	0.047	0.03
(B10) Mo 0.2% + CMC	0.007	0.003	0.014	0.026	0.008	0.008	0.024	0.016	0.013
(B11) Mo 0.2% + AE	0.011	0.008	0.025	0.021	0.007	0.019	0.014	0.023	0.021
(B12) Mo 0.2% + AES	0.035	0.028	0.017	0.019	0.024	0.005	0.012	0.018	0.006
