# Supplementary material for: Molybdate in Rhizobial Seed-Coat Formulations Improves the Production and Nodulation of Alfalfa
Source: PLoS One. 2017 Jan 18;12(1):e0170179. doi: 10.1371/journal.pone.0170179 (PMC5242510; doi:10.1371/journal.pone.0170179)
Supplement: S9 Table — (PDF) [file pone.0170179.s009.pdf]

S9 Table. The nitrogenase activity of root nodules in alfalfa inoculated with ACCC17676 rhizobia seed-coat formulation.

Nitrogenase activity(μmol ml^{-1} h^{-1})	Rpt.1	Rpt.2	Rpt.3
(B1) Mo 0% + CMC	49.67993	22.3729	43.00436
(B2) Mo 0% + AE	50.70303	42.37685	72.7623
(B3) Mo 0% + AES	75.69971	95.27449	67.88151
(B4) Mo 0.05% + CMC	122.286	114.5838	39.28504
(B5) Mo 0.05% + AE	97.03744	123.5897	117.989
(B6) Mo 0.05% + AES	210.9138	139.8952	164.419
(B7) Mo 0.1% + CMC	197.1553	206.8037	181.2571
(B8) Mo 0.1% + AE	219.6874	137.1469	245.3324
(B9) Mo 0.1% + AES	257.6577	265.4243	308.9147
(B10) Mo 0.2% + CMC	6.210109	5.429481	10.02552
(B11) Mo 0.2% + AE	45.50176	4.479177	5.677994
(B12) Mo 0.2% + AES	7.214587	13.04826	10.08131
